# Association Between Depression and Polypharmacy in Older Adults—A Systematic Review and Meta‐Analysis

**DOI:** 10.1002/jgf2.70094

**Published:** 2026-01-04

**Authors:** Yi‐Hsin Wang, Tsung‐Yu Tsai, Hung‐Chang Chou, Huei Chung

**Affiliations:** ^1^ Department of Psychiatry MacKay Memorial Hospital Taipei Taiwan; ^2^ Department of Epidemiology, Gillings School of Global Public Health University of North Carolina at Chapel Hill Chapel Hill North Carolina USA; ^3^ Department of Pharmacy and Master Program Tajen University Pingtung Taiwan; ^4^ Department of Pharmacy Taoyuan General Hospital, Ministry of Health and Welfare Taoyuan Taiwan

**Keywords:** depression, older adults, polypharmacy

## Abstract

**Background:**

Depression and polypharmacy are both common among older adults and may be closely interrelated.

**Methods:**

This systematic review and meta‐analysis examined the association between depression and polypharmacy by searching EMBASE, PubMed, Web of Science, and PsycINFO from inception to 18 May 2023. The review was registered in PROSPERO (CRD42022343619).

**Results:**

Eight studies met the inclusion criteria, with six rated high quality and two medium quality. Pooled analysis using a random‐effects model demonstrated a strong association between depression and polypharmacy (OR = 2.49; 95% CI: 1.88–3.29; I² = 83%). Subgroup analyses revealed consistently elevated associations in both community‐based and institutional/clinic‐based populations.

**Conclusions:**

Depression is associated with polypharmacy among older adults. These findings underscore the importance of integrating mental health assessment into medication management strategies for older adults.

## 1 Introduction

According to the Global Burden of Disease Study [1], depression ranked as the 13th leading cause of disability‐adjusted life years and the 2nd highest contributor to years lived with disability in 2019. In the context of rapid demographic aging, depression not only contributes substantially to the global disease burden but also exerts disproportionate effects on older populations [2]. As reported by the World Health Organization (WHO) [3], approximately 7% of the global elderly population suffers from depressive disorders. A systematic review [4] of 42 studies conducted worldwide between 1994 and 2019 further revealed that 31.74% of older adults reported depressive symptoms. Chronic physical illnesses—such as heart disease and persistent pain—exacerbate vulnerability to geriatric depression by intensifying both physiological and psychosocial stressors. Moreover, depression increases the risk of receiving suboptimal physical healthcare and heightens perceptions of social isolation, thereby perpetuating a vicious cycle of deteriorating physical and mental health in later life [5, 6, 7, 8].

The World Health Organization (WHO) defines polypharmacy as the administration of multiple or an excessive number of medications. In contemporary research, it is most commonly operationalized as the concurrent use of five or more medications [9, 10]. A large‐scale study [11] involving 1.7 million Swedish individuals aged over 65, reported a polypharmacy prevalence of 44.0%, with 11.7% affected by excessive polypharmacy (≥ 10 medications). Polypharmacy has been associated with a wide range of adverse outcomes, including hospitalization, drug–drug interactions, frailty, falls, and mortality [12, 13, 14, 15, 16, 17, 18]. It also contributes to various physical and psychological complications: a Finnish study [19] found that taking four or more medications significantly increased urinary symptoms; a 12‐year Korean cohort study [20] linked polypharmacy to dementia onset, and the use of 10 or more drugs was associated with nutritional decline [21]. Furthermore, polypharmacy was found to increase the risk of depression by 73%, with higher medication counts correlating with poorer trajectories of late‐life depression [22].

Although several studies have suggested that polypharmacy may increase the risk of depression, the bidirectional relationship between these two conditions remains inconclusive. Although some evidence points to a negative association between depressive symptoms and medication use, others report a significant positive correlation [23, 24]. Despite growing research interest, it is still unclear whether depression contributes to an elevated risk of polypharmacy. Therefore, this study aimed to examine whether depression is associated with a higher likelihood of developing polypharmacy among older adults.

## 2 Methods

### 2.1 Search Strategy

This systematic literature review and meta‐analysis were conducted in accordance with the PRISMA (Preferred Reporting Items for Systematic Reviews and Meta‐Analyses) guidelines. A comprehensive search was conducted across four major databases—PubMed, Web of Science, Embase, and PsycINFO—from their inception to 18 May 2023. These databases were selected for their broad coverage and relevance to geriatric psychiatry, mental health, and pharmacoepidemiology [25, 26]. PubMed, EMBASE, and Web of Science were used to initially assemble the dataset. After subsequently adding PsycINFO, no additional eligible studies were identified. Standardized terms and keywords were used to search for the following core concepts: old age, polypharmacy, and depression. No restrictions were applied regarding publication date, language, or study design (see Appendix S1). In addition, the reference lists of relevant articles were manually screened to identify additional eligible studies. All retrieved records were exported to EndNote (Thomas Reuters, New York, NY, USA), where duplicates were identified and removed. The study protocol was registered with.

### 2.2 Selection Criteria

Studies were included if they met the following criteria: (1) involved middle‐aged and older adults aged 60 years or above, a commonly used threshold in aging research; (2) defined polypharmacy as the use of five or more medications; and (3) were clinical trials, observational cohort studies, or cross‐sectional studies that assessed the risk of polypharmacy in individuals with depressive symptoms. The studies were excluded if they: (1) evaluated depressive symptoms as the exposure predicting outcome, medication use or polypharmacy; (2) focused on patients with serious medical or physical conditions or those who were hospitalized; (3) duplicated data from previously published articles; (4) were non‐original research formats such as editorials, posters, conference proceedings, or case reports; or (5) applied inappropriate definitions of the population, exposure, or outcome—such as including participants under 60 years of age or defining polypharmacy as fewer than five medications.

### 2.3 Data Extraction

A pre‐defined data extraction template was developed by Y.‐H.W. to capture key study characteristics, including first author, year of publication, title, study design, sample size, age criteria, sex distribution, diagnostic criteria for depression, definition of polypharmacy, risk estimates with corresponding 95% confidence intervals (CIs), covariates controlled, and inclusion/exclusion criteria. Two independent reviewers (Y.‐H.W. and T.‐Y.T.) assessed the full text of each eligible article and extracted the relevant data. If consensus could not be reached, additional input was sought from other authors (H.C. and H.‐C.C.).

### 2.4 Quality Appraisal

Study quality was assessed using a modified version of the Newcastle‐Ottawa Quality Assessment Scale (NOS) [27], adapted to evaluate both cross‐sectional and cohort studies. One author (Y.‐H.W.) conducted the initial quality assessment, and any uncertainties were resolved through discussion with a second reviewer (T.‐Y.T.). Each cross‐sectional study was scored on an 8‐point scale, and each cohort study on a 9‐point scale. The NOS evaluates three domains: (1) methodological quality (maximum of 4 points), (2) study comparability (2 points), and (3) outcome assessment (2 points for cross‐sectional and 3 points for cohort studies). Based on these criteria, studies were classified as having good (6–9 stars), fair (3–5 stars), or poor (0–2 stars) quality [28].

### 2.5 Data Synthesis and Statistical Analysis

All estimates—including prevalence data, odds ratio (OR), and risk ratio (RR)—were extracted to evaluate the association between depression and polypharmacy, and subsequently converted into ORs for consistency. For studies that reported polypharmacy and excessive polypharmacy separately (defined as the concurrent use of more than 9 or 10 medications), data were harmonized to generate unified ORs. Likewise, when depression severity was categorized into multiple levels, these were consolidated into two groups—depressed and non‐depressed—for statistical analysis. When available, adjusted odds ratios were used; otherwise, crude ORs were calculated from the original data.

Random‐effects models with inverse variance weighting were applied using Review Manager (RevMan 5.4.2; The Nordic Cochrane Centre, Copenhagen, Denmark). Heterogeneity across studies was assessed using the *Q* statistic and the *I* ^2^ statistic [29]. An *I* ^2^ value below 30% was considered indicative of low heterogeneity, whereas values above 60% were interpreted as substantial heterogeneity. A funnel plot (Appendix S2) was generated to visually assess potential publication bias; however, formal tests for funnel‐plot asymmetry were not performed because fewer than 10 studies limited the reliability of such assessment [30]. Where appropriate, subgroup analyses were carried out to explore potential sources of heterogeneity. The certainty of evidence for each major outcome was evaluated using the Grading of Recommendations Assessment, Development and Evaluation (GRADE) approach, with ratings categorized as high, moderate, low, or very low [31].

## 3 Results

### 3.1 Study Selection

**FIGURE 1:**
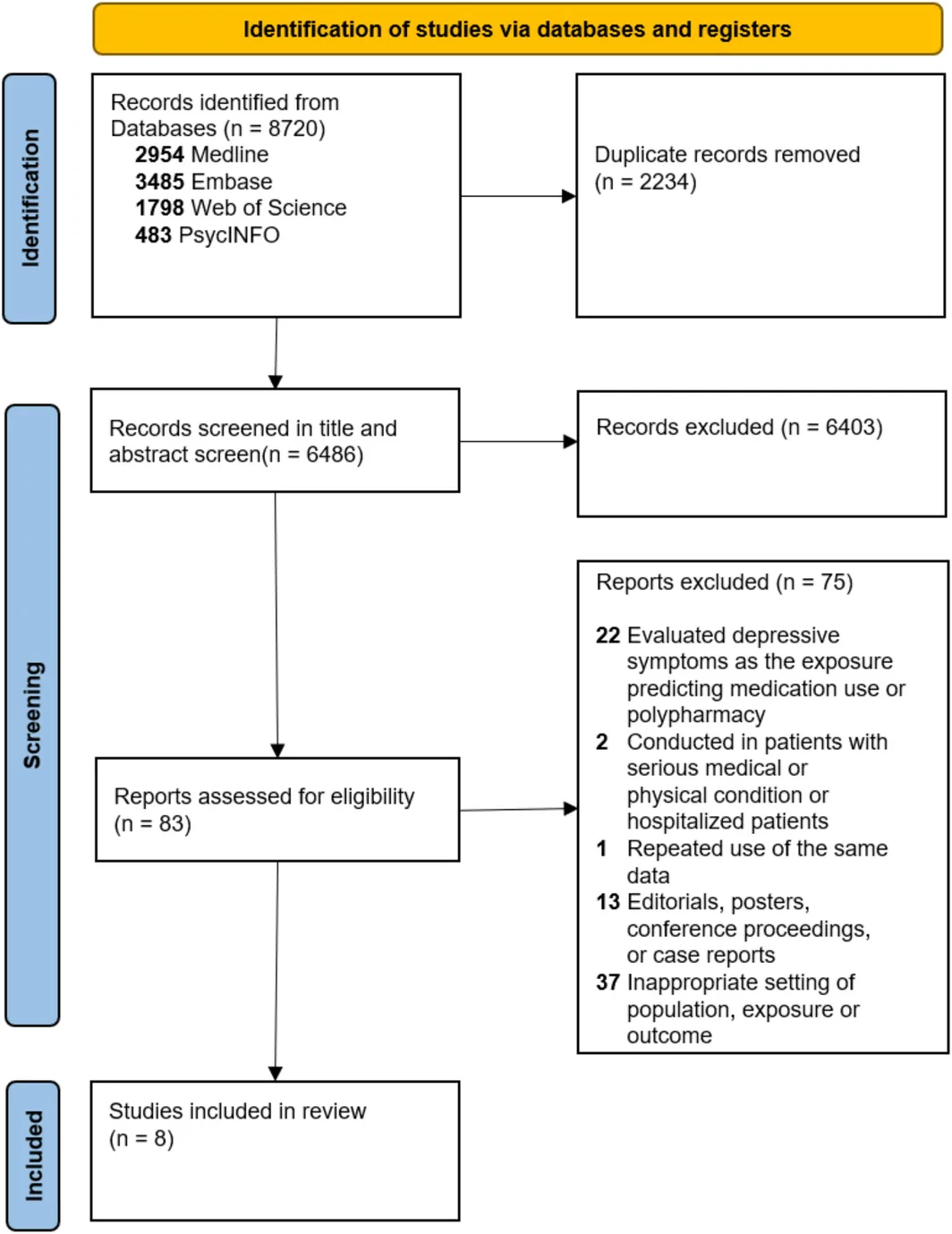
PRISMA diagram for the systemic review of the association between depressive symptoms and polypharmacy.

The initial search yielded 8720 records, with 2234 duplicates removed, leaving 6486 for screening. After title and abstract review, 6403 were excluded. Eighty‐three full‐text articles were assessed, of which 75 were excluded for reasons including reverse exposure–outcome direction (*n* = 22), inappropriate populations (*n* = 2), data overlap (*n* = 1), missing full texts (*n* = 13), and unsuitable definitions of depression or polypharmacy (*n* = 37). Ultimately, seven studies were included in the analysis. The PRISMA flow diagram was presented in Figure 1.

### 3.2 Study Characteristics

Eight articles [23, 32, 33, 34, 35, 36, 37] published between 2012 and 2023 included 164–4156 participants across 10 countries (Czech Republic, England, Finland, France, Germany, Italy, The Netherlands, Belgium, Turkey, Saudi Arabia, and Taiwan). Table 1 summarized the characteristics of these studies. Seven were cross‐sectional, and one was a longitudinal observational study. Three studies enrolled participants aged ≥ 60, three focused on those ≥ 65, and one targeted individuals > 80. One nursing home study was included despite lacking a clear age cutoff.

**TABLE 1:** Studies examined the association between PP and depressive symptoms.

Authors	Study design	Location and year of study	Sample size^a^	Age^b^	Depression criteria	PP criteria	Methods of obtaining medication information	Outcome
Onder, 2012	Cross‐sectional study	Nursing home residents, 2009–2011	4023 (1078/2945)	83.5 ± 9.4	Major Depression Rating Scale ≥ 3	PP: 5–9, EPP: ≧ 10	Researchers collected, 3 days prior to the assessment	PP OR: 1.43 (1.17–1.75) EPP OR: 1.81 (1.38–2.37)
Walckier, 2015	Cross‐sectional study	Community‐based and institutionalized residents, 2008.	2845 (1172/1673)	≧ 65	Self‐completion questionnaire “depression that has lasted for at least 2 weeks during the past 12 months”	PP: 5–8, EPP: ≧ 9	Researchers collected, 24 h prior to the assessment	PP OR: 1.54 (1.02–2.33) EPP OR: 3.48 (2.03–5.97)
Wauters, 2016	Cross‐sectional study	Community‐based residents, 2008–2009	503 (195/308)	≧ 80	GDS‐15 FOR SCREEN and CONFIRMED by general practitioners according to clinical impression	PP: ≧ 5 EPP: ≧ 10	Researchers collected	OR: 3.7 (1.4–9.7)
Yuruyen, 2016	Cross‐sectional study	Hospital‐based patients, 2003–2012	1205 (351/854)	≧ 65	Researchers reviewed hospital record who presented to the geriatric clinic (no clarified definition)	PP: ≧ 5 EPP: ≧ 9	Researchers collected (patients received multidimensional geriatric assessment that included re‐evaluation of polypharmacy)	OR:4.5 (3.2–6.5)
Ersoy, 2019	Cross‐sectional study	Community‐based residents, 2014–2017	707 (265/442)	≧ 65	Geriatric Depression Scale ≧ 14 (Geriatric Depression Scale 30 items)	PP: ≧ 5	Research collected, only continuously consumed medicines were taken into consideration (antibiotics and temporary analgesics were neglected)	OR:1.66 (1.19–2.29)
Wiersema, 2022	Longitudinal observational study	Community‐based patients, 2007–2010	507 (179/328)	≧ 60	2‐year follow‐up, according to the Diagnostic and Statistical Manual of Mental Disorders‐IV‐R criteria	PP: ≧ 5	Research collected (Dermatological preparations, medications without an ATC code, medications used less than half of the week were excluded)	Chi‐square statistic: 30.2 df = 1, *p* < 0.001
Aljawadi, 2022	Cross‐sectional study	Community‐based residents, 2006–2007	2946 (1759/1187)	≧ 60	Geriatric Depression Scale ≧ 5–10 (suggestive) 11–15 (depression) (Geriatric Depression Scale15 items)	PP: ≧ 5	Research collected (household visit, 6 month prior to the assessment)	RR of GDS 5–10: 1.379 (1.259–1.512) RR of GDS 11–15: 1.253 (0.964–1.629)

Abbreviations: EPP, excessive polypharmacy; GDS, geriatric depression scale; OR, odds ration; PP, polypharmacy; RR, risk ratio.

^a^ Total sample size, male/female.

^b^ Median or mean, SD (range if available).

### 3.3 Quality and Publication Bias Appraisal

Among the eight included articles, six were rated as good and two as fair (Table 2). One study [23] lacked sample representativeness because of non‐random selection, and another [34] relied solely on a self‐administered questionnaire to define depression, raising concerns about diagnostic accuracy. None of the studies addressed non‐respondents. For comparability, two articles [32, 38] did not adjust for confounding via multivariate analysis. All studies clearly reported medication data collection methods and applied appropriate statistical analyses.

**TABLE 2:** Risk of bias assessment by the Newcastle‐Ottawa Assessment scale.

Cross‐sectional study								
Author, year	Selection (maximum 4)				Comparability (maximum 2)	Outcome (maximum 2)		Total
	Representativeness of the sample	Sample size	Ascertainment of the exposure	Non‐respondents		Assessment of the outcome	Statistical test	(*/8)
Onder, 2012	^—^	^*^	^*^	^—^	^**^	^*^	^*^	6
Walckier, 2015	^*^	^*^	^—^	^—^	^**^	^*^	^*^	6
Wauters, 2016	^*^	^*^	^*^	^—^	^**^	^*^	^*^	7
Yuruyen, 2016	^*^	^*^	^*^	^—^	^**^	^*^	^*^	6
Ersoy, 2019	^*^	^*^	^*^	^—^	^—^	^*^	^*^	5
Aljawadi, 2022	^*^	^*^	^*^	^—^	^**^	^*^	^*^	7

Cohort study									
Author, year	Selection (maximum 4)				Comparability (maximum 2) of cohorts on the basis of the design or analysis	Outcome (maximum 3)			Total
	Representativeness of the cohort	Selection of the non‐exposed cohort	Ascertainment of the exposure	Demonstration that outcome of interest was accounted for or not present at start of study		Assessment of the outcome	Was follow‐up long enough or outcomes to occur	Adequacy of follow‐up of cohorts	(*/9)
Wiersema, 2022	^*^	^*^	^*^	^*^	^**^	^*^	^*^	^*^	9

^*^ Indicates that the study met the corresponding Newcastle–Ottawa Scale (NOS) criterion and was awarded one point.

^**^ In the comparability domain indicate that two points were awarded. Specifically, studies received two points when multivariable regression analyses were used to control for potential confounders, whereas studies using only univariable or simple statistical approaches (e.g., chi‐square tests) received one or zero points, as appropriate.

^—^ Indicates that the criterion was not met and no point was assigned.

### 3.4 Outcome Measure and Meta‐Analysis

Across all included studies, depressive symptoms were consistently associated with an elevated risk of polypharmacy. Among older adults with or without depression, the prevalence of polypharmacy ranged from 32.8% to 92.7%, whereas excessive polypharmacy ranged from 8.2% to 26.2%.

To further support the robustness of this association, one study [37] employed multivariable regression analysis and identified depression as an independent predictor of polypharmacy, even when compared with other comorbid conditions. Another study [36] conducted a sensitivity analysis excluding psychotropic medications and confirmed that the association between depressive symptoms and polypharmacy persisted. Although various factors—such as socioeconomic status (e.g., older age, geographic region, and income) and functional status—may contribute to increased medication use, the evidence supports a consistent association between depression and polypharmacy.

**FIGURE 2:**
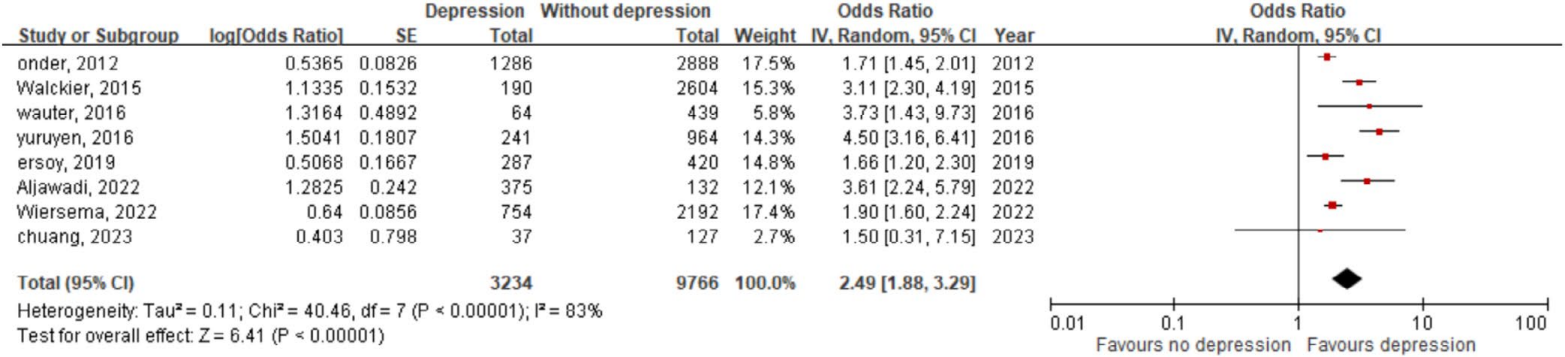
Forest plot of the association between depressive symptoms and polypharmacy. df, degree of freedom; IV, inverse variance; SE, standard error.

A meta‐analysis was conducted to evaluate the association between depression and polypharmacy, without assuming a directional relationship. As shown in Figure 2, the pooled OR was 2.49 (95% CI: 1.88–3.29, *p* < 0.001). Substantial heterogeneity was observed (*I* ^2^ = 83%, *p* < 0.00001). The corresponding 95% prediction interval was 1.56–4.11.

**FIGURE 3:**
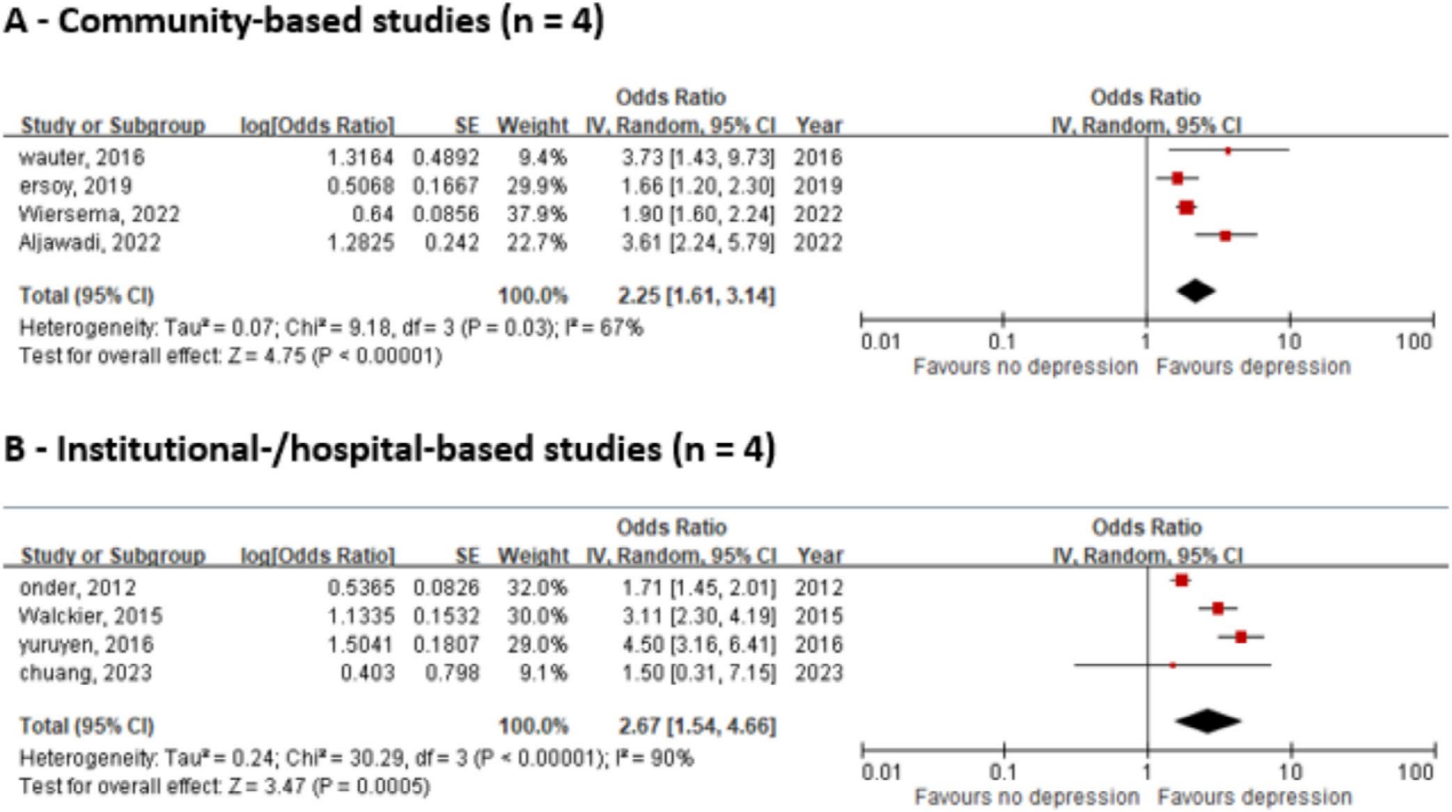
Subgroup analyses of the association between depressive symptoms and polypharmacy by care setting. df, degree of freedom; IV, inverse variance; SE, standard error.

To further explore this heterogeneity, a subgroup analysis was performed based on care setting (Figure 3). Four studies were classified as community‐based, whereas the remaining four were categorized as institutional‐/hospital‐based, involving more clinically complex populations from institutional or hospital settings. Both subgroups demonstrated elevated odds of polypharmacy: the community‐based group yielded an OR of 2.25 (1.61–3.14; *I* ^2^ = 67%; Figure 3A), and the institutional–/clinic‐based group yielded an OR of 2.67 (1.54–4.66; *I* ^2^ = 90%; Figure 3B).

The GRADE assessment of the certainty of evidence regarding the association between depression and polypharmacy is presented in Appendix S3. Overall, the evidence was rated as low, primarily because of the observational design of most included studies and the substantial heterogeneity observed in the meta‐analysis results.

## 4 Discussion

This systematic review and meta‐analysis aimed to comprehensively quantify the association between depression and polypharmacy in older adults. A total of eight studies comprising 13,000 participants were included. The pooled analysis indicated that individuals aged over 60 with depressive symptoms had significantly higher odds of experiencing polypharmacy, with a pooled odds ratio of 2.49. These findings underscore a consistent and clinically meaningful association between depression and increased medication burden in later life.

A majority of the included studies demonstrated a positive association between depressive symptoms and polypharmacy, with 7 out of 8 studies reporting consistent findings. Among these, two included articles [32, 37] explored this issue from a chronic disease perspective, emphasizing that depression often necessitates pharmacological treatment and frequently coexists with other independent risk factors for increased medication use, such as metabolic syndrome, chronic pain, urinary incontinence, elevated creatinine levels, and gastric disturbances. Although depressive symptoms are rarely directly classified as medication side effects, a bidirectional relationship may exist, with polypharmacy and depression potentially reinforcing each other in a self‐perpetuating cycle. One study [39] reported that more severe depressive symptoms were associated with greater medication use. Another study [34] found that higher levels of medication use were linked to a more severe course of late‐life depression, implying that polypharmacy itself may exacerbate depressive outcomes. This pattern may also help explain why depression was identified as an independent predictor of polypharmacy in another analysis [35], potentially exerting a stronger influence than other comorbid conditions [37].

Some studies not focused on older adults have reported a negative correlation between depressive symptoms and the number of medications used; however, methodological limitations should be taken into consideration. One longitudinal study [24] examined the relationship between obesity and depression, reporting a reduced need for medication at follow‐up, as depressive symptoms present at baseline had been effectively treated and resolved. Another study [40] found no positive association after adjusting for chronic diseases, which may be attributable to the lower threshold used to define polypharmacy, potentially resulting in a ceiling effect that limited the detection of differences between individuals with and without depressive symptoms.

This review offers several methodological strengths. It is the first to rigorously investigate the association between depressive symptoms and polypharmacy, specifically in older adults, utilizing a prespecified analytic framework, a standardized definition of polypharmacy (≥ 5 medications), and an age‐restricted population (≥ 60 years). The inclusion of a psychiatric‐focused database enhanced the comprehensiveness of mental health literature coverage. In addition, several subgroup analyses were conducted to evaluate the robustness of findings and identify potential sources of heterogeneity. Nonetheless, several limitations should be acknowledged. Most included studies (7 of 8; 88%) used cross‐sectional designs, limiting the ability to infer temporal sequence or causality. The potential for selection bias cannot be excluded. None of the included studies reported the number or characteristics of non‐respondents. Individuals in poorer health or with more severe depressive symptoms may be less likely to participate in surveys, potentially leading to an underestimation or distortion of the true association. Also, substantial between‐study heterogeneity may reflect differences in participant recruitment, variability in the assessment of depression and medication use, and inconsistencies in covariate adjustment, all of which may affect comparability and limit the generalizability of pooled estimates.

## 5 Conclusion

This review highlights the association between depression and an increased risk of polypharmacy. Given that both conditions are prevalent among older adults, the findings underscore the importance of carefully evaluating depressive symptoms prior to initiating pharmacological treatment in geriatric patients.

## Author Contributions

**Tsung‐Yu Tsai:** conceptualization, methodology, formal analysis, writing and original draft, writing and review editing, supervision. **Hung‐Chang Chou:** writing and review editing. **Huei Chung:** writing and review editing.

## Funding

The authors have nothing to report.

## Conflicts of Interest

The authors declare no conflicts of interest.

## Supporting information


**Appendix S1:** Search strategies for meta‐analysis.
**Appendix S2:** Funnel plot of adjusted association between depressive symptoms and polypharmacy.
**Appendix S3:** Summary of findings and quality of evidence.

## Data Availability

The data that support the findings of this study are available from the corresponding author upon reasonable request.
