# Supplementary material for: Association Between Depression and Polypharmacy in Older Adults—A Systematic Review and Meta‐Analysis
Source: J Gen Fam Med. 2026 Jan 4;27(1):e70094. doi: 10.1002/jgf2.70094 (PMC12897565; doi:10.1002/jgf2.70094)
Supplement: Supplementary file 1 — Appendix S1: Search strategies for meta‐analysis. Appendix S2: Funnel plot of adjusted association between depressive symptoms and polypharmacy. Appendix S3: Summary of findings and quality of evidence. [file JGF2-27-e70094-s001.docx]

**Appendix S1: Search strategies for meta-analysis**

Database: Embase

Search Strategy:

--------------------------------------------------------------------------------------------------------------------------------

1 (dysthmi* OR depressi* OR 'mood disorder*' OR 'affective disorder*'):ti,ab,kw,de

2 “depression”/exp

3 aged:ti,ab,kw,de OR elder*:ti,ab,kw,de OR geriatric*:ti,ab,kw,de OR gerontolog*:ti,ab,kw,de OR senior*:ti,ab,kw,de OR senium*:ti,ab,kw,de OR pensioner*:ti,ab,kw,de OR 'old age' OR ((older NEAR/2 (adult* OR person* OR inpatient* OR outpatient* OR population* OR men OR women OR male OR female OR subject OR citizen*)):ti,ab,kw,de)

4. "aged"/exp OR "Senescence”/exp

5 polypharm*:ti,ab,kw,de OR (((multi* OR concomitant OR concurrent OR poly) NEXT/2 (medication* OR drug OR pharm* OR regimen*)):ti,ab,kw,de)

6 ”polypharmacy”/exp

7. (#1 OR #2) AND (#3 OR #4) AND (#5 OR #6) AND [embase]/lim

--------------------------------------------------------------------------------------------------------------------------------

Database: PsycINFO

Search Strategy:

--------------------------------------------------------------------------------------------------------------------------------

1 (dysthmi* OR depressi* OR 'mood disorder*' OR 'affective disorder*'):ti,ab,kw,de

2 polypharmacy* OR ((Multi* OR concomitant OR concurrent OR poly) w2 (medication* OR drug* OR pharm* OR regimen*))

3 aged OR elder* OR geriatric* OR gerontolog* OR senior* OR senium* OR pensioner* OR "old age*" OR (older n2 (adult* OR person* OR people OR patient* OR inpatient* OR outpatient* OR population* OR men OR women OR male OR female OR subject* OR citizen*))

4 1 and 2 and 3

--------------------------------------------------------------------------------------------------------------------------------

Database: Medline

Search Strategy:

--------------------------------------------------------------------------------------------------------------------------------

1 (dysthmi* or depressi* or 'mood disorder*' or 'affective disorder*').ti,ab,kf,de.

2 exp "Depressive Disorder"/ or exp "Depression"/

3 (aged or elder* or geriatric* or gerontolog* or senior* or senium* or pensioner* or "old age*").mp. or (older adj2 (adult* or person* or people or patient* or inpatient* or outpatient* or population* or men or women or male or female or subject* or citizen*)).ti,ab,kf,de.

4 exp "aged"/

5 (Polypharm* or ((Multi* or concomitant or concurrent or poly) adj2 (medication* or drug* or pharm* or regimen*))).ti,ab,kf,de.

6 exp "polypharmacy"/

7 (1 or 2) and (3 or 4) and (5 or 6)

--------------------------------------------------------------------------------------------------------------------------------

Database: Web of Science

Search Strategy:

--------------------------------------------------------------------------------------------------------------------------------

1 ts=(dysthmi* OR depressi* OR 'mood disorder*' OR 'affective disorder*')

2 ts=(aged OR elder* OR geriatric* OR gerontolog* OR senior* OR senium* OR pensioner* OR "old age*" OR (older NEAR/2 (adult* OR person* OR people OR patient* OR inpatient* OR outpatient* OR population* OR men OR women OR male OR female OR subject* OR citizen*)))\

3 tS=(Polypharm* OR ((Multi* OR concomitant OR concurrent OR poly) NEAR/2 (medication* OR drug* OR pharm* OR regimen*)))

4 1 and 2 and 3


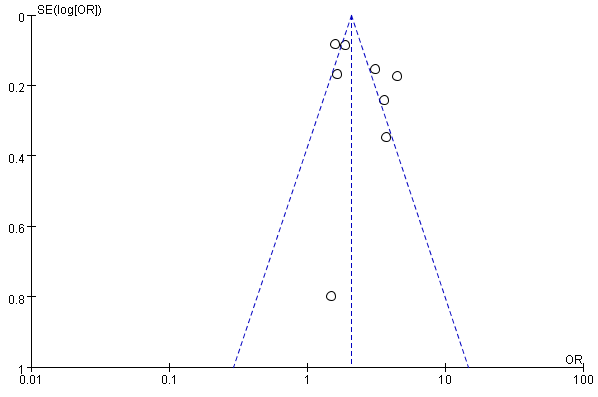
**Appendix S2. Funnel plot of adjusted association between depressive symptoms and polypharmacy. OR: odds ratio; SE: standard error**

Appendix S3. Summary of Findings and Quality of Evidence

| Outcome | Study Design | Findings | Study Limitations | Inconsistency | Indirectness | Imprecision | GRADE |
| --- | --- | --- | --- | --- | --- | --- | --- |
|  |  |  |  |  |  |  |  |
| Polypharmacy (≥5 medications) | 7 observational studies; 12,836 participants | OR 2.53 (95% CI: 1.90–3.36), favors association between depressive symptoms and polypharmacy | Observational design; some studies unclear in exposure and outcome assessment (downgraded) | Substantial heterogeneity (I² = 85%) | Direct (no serious indirectness) | Precise estimate; CI excludes null | Low |
| NOTE: OR=odds ratio; CI=Confidence intervals | | | | | | | |
